# The Elusive Coreceptors for the SARS-CoV-2 Spike Protein

**DOI:** 10.3390/v15010067

**Published:** 2022-12-25

**Authors:** Reed L. Berkowitz, David A. Ostrov

**Affiliations:** Department of Pathology, Immunology and Laboratory Medicine, University of Florida College of Medicine, Gainesville, FL 32610, USA

**Keywords:** SARS-CoV-2, COVID-19, N-terminal domain, receptor binding domain, LRRC15, leucine rich repeat containing 15, neuropilin-1, NRP-1, mutations

## Abstract

Evidence suggests that the N-terminal domain (NTD) of the SARS-CoV-2 spike protein interacts with host coreceptors that participate in viral entry. Resolving the identity of coreceptors has important clinical implications as it may provide the basis for the development of antiviral drugs and vaccine candidates. The majority of characteristic mutations in variants of concern (VOCs) have occurred in the NTD and receptor binding domain (RBD). Unlike the RBD, mutations in the NTD have clustered in the most flexible parts of the spike protein. Many possible coreceptors have been proposed, including various sugars such as gangliosides, sialosides, and heparan sulfate. Protein coreceptors, including neuropilin-1 and leucine-rich repeat containing 15 (LRRC15), are also proposed coreceptors that engage the NTD.

## 1. Introduction to Coronavirus Spike Proteins (Function, Structure, Receptors)

### 1.1. Function and Structure

The coronavirus spike (S) protein, comprised of S1 and S2 subunits, mediates the entry of coronaviruses into host cells. Generally, in the viral attachment of coronaviruses, the S1 subunit binds to a receptor on the host cell’s surface. This binding prompts a conformational change in the spike protein that enables the S2 subunit to fuse together the viral and host membranes [1].

Due to its fundamental role in initiating infection, the S protein is of high relevance to natural immune response as well as clinical therapies. It follows that SARS-CoV-2 infection elicits neutralizing antibodies that bind to the S protein, and that vaccines targeting the S protein provide protection against infection.

Each spike protein monomer is comprised of approximately 1273 residues (depending on deletions in certain variants) which trimerize upon assembly into a clove-shaped trimer with three S1 heads and a trimeric S2 stalk [2]. There are characteristic domains present in all coronavirus spike domains. In the SARS-CoV-2 S1, these include the N-terminal domain (NTD, residues 14–305) and receptor-binding domain (RBD, residues 319–541). In S2, these include the fusion peptide (FP, residues 788–806), heptad repeat sequences 1 (HR1, residues 912–984) and 2 (HR2, residues 1163–1213), transmembrane domain (TM, residues 1213–1237), and intracellular domain (IC, residues 1237–1273) [2].

### 1.2. Receptors

Different coronaviruses exhibit a diverse range of tropism to different cells and tissues, resulting in varying clinical manifestations ranging from mild to severe disease (e.g., HCoV-229E results in the common cold while MERS-CoV results in approximately 35% mortality) [3]. S1 C-terminal domains (CTDs) are known to bind to protein receptors, such as ACE2 in SARS-CoV and SARS-CoV-2 or dipeptidyl peptidase 4 (DPP4) in MERS-CoV. The S1-NTD has been shown to bind host sugar moieties for the majority of coronaviruses (e.g., TGEV and PEDV) [1]. A subset of coronaviruses exhibits NTDs that bind protein elements (e.g., the NTD of mouse hepatitis virus, MHV, binds carcinoembryonic antigen-related cell adhesion molecule 1, CEACAM1) [1]. Recent studies have suggested a dual-receptor mechanism for MERS-CoV, positing that it binds not only to DPP4 at the CTD but also to host sugars (sialosides) at the NTD [4].

The host cell receptor for the NTD of SARS-CoV-2 is not yet known [5]. Resolving the identity of such a ligand could have a large impact on drug development and COVID treatment strategies. This unidentified ligand could either act as a coreceptor that helps facilitate entry mediated by the primary receptor (e.g., CCR5 and CD4 for HIV) or a distinct alternative receptor (e.g., CD147 for SARS-CoV-2) [6,7]. Since the putative ligands of the SARS-CoV-2 S1-NTD are not clear, it is also not clear how mutations in the S1-NTD might affect virus fitness.

### 1.3. Mutations in Variants of Concern (VOCs)

**Figure 1:**
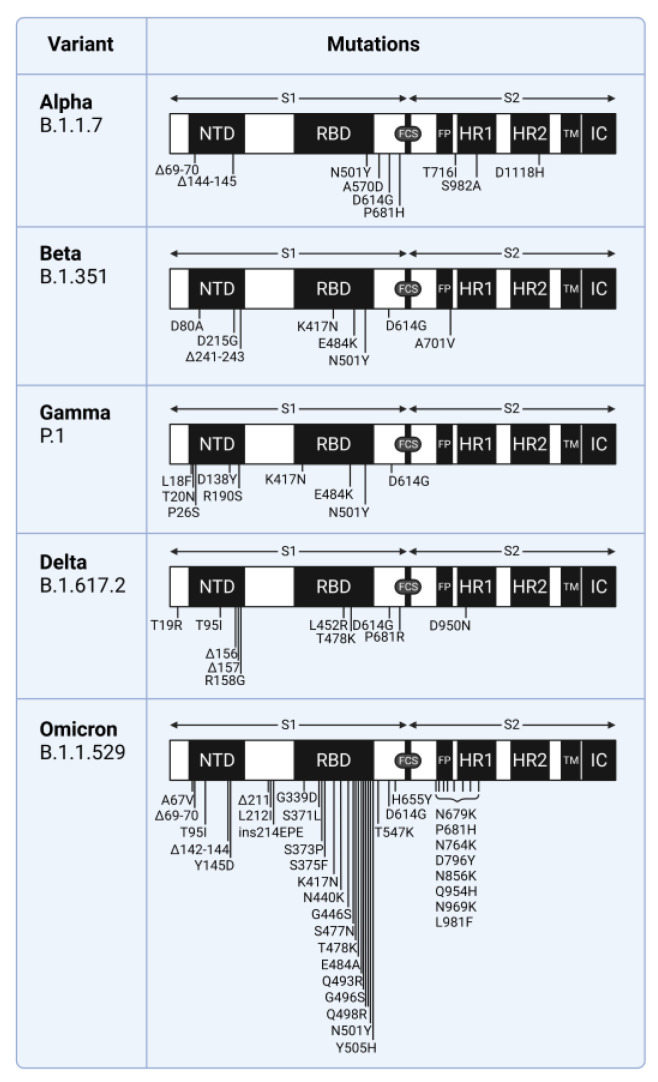
Mutations in the SARS-CoV-2 spike glycoprotein. NTD, N-terminal domain; RBD, receptor binding domain; FCS, furin cleavage site; FP, fusion peptide; HR1, heptad repeat 1; HR2, heptad repeat 2; TM, transmembrane anchor; IC, intracellular tail. The Delta symbol indicates deletion. Mutations as defined by the Centers for Disease Control and Prevention.

**Figure 2:**
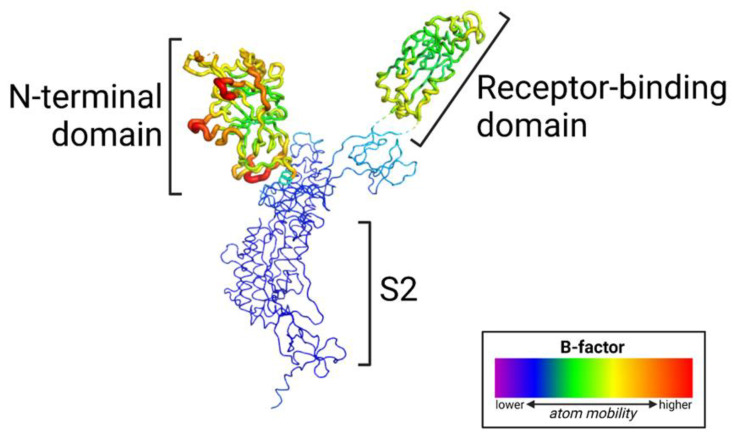
The N-terminal domain (NTD) of the SARS-CoV-2 spike glycoprotein exhibits a high level of flexibility. The cryo-EM structure of the prefusion spike protein is colored by B-factor; the thickness of the wire corresponds to structural variability.

In variants of concern (VOC) of SARS-CoV-2, many distinguishing mutations of the spike protein occur at the S1-NTD and S1-RBD (Figure 1). The majority of S1-NTD mutations that distinguish VOCs occur on the solvent-accessible surface at a site available for binding ligands (Figure 2). Additionally, SARS-CoV-2 elicits neutralizing antibodies binding to multiple locations of the NTD [5,8]. These facts suggest that the NTD has some important but unknown functions affecting virus fitness, such as coreceptor binding, avoidance of restriction factors, or antibody-dependent enhancement [8]. Specifically, host cell restriction factors might block virus entry by binding the S1-NTD, so mutations here could help the virus evade this defense.

**Figure 3:**
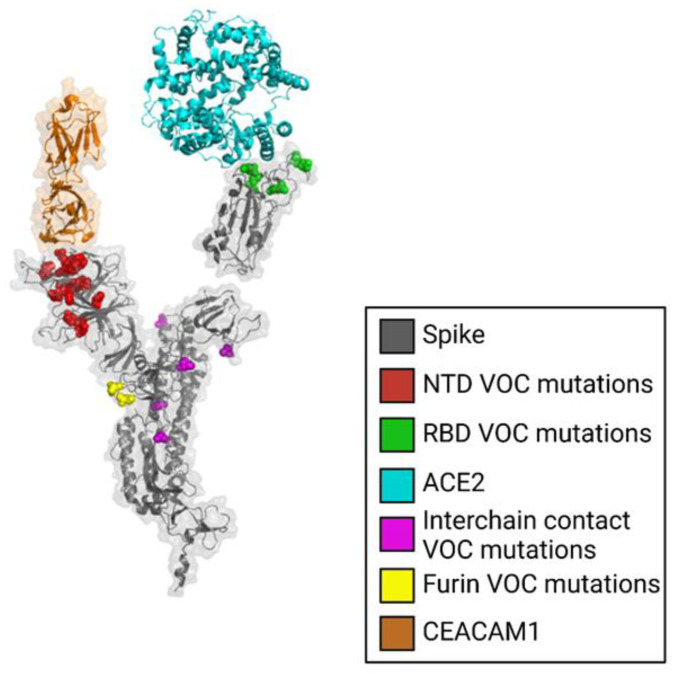
Mutations in the N-terminal domain (NTD) cluster in a manner that likely modulates binding interactions. The spike glycoprotein is shown in gray with mutations present in variants of concern (VOC) shown in red, NTD VOC mutations in red, RBD VOC mutations in green, interchain contact VOC mutations in magenta, furin cleavage site mutations shown in yellow, ACE2 shown in cyan, CEACAM1 shown in orange.

**Figure 4:**
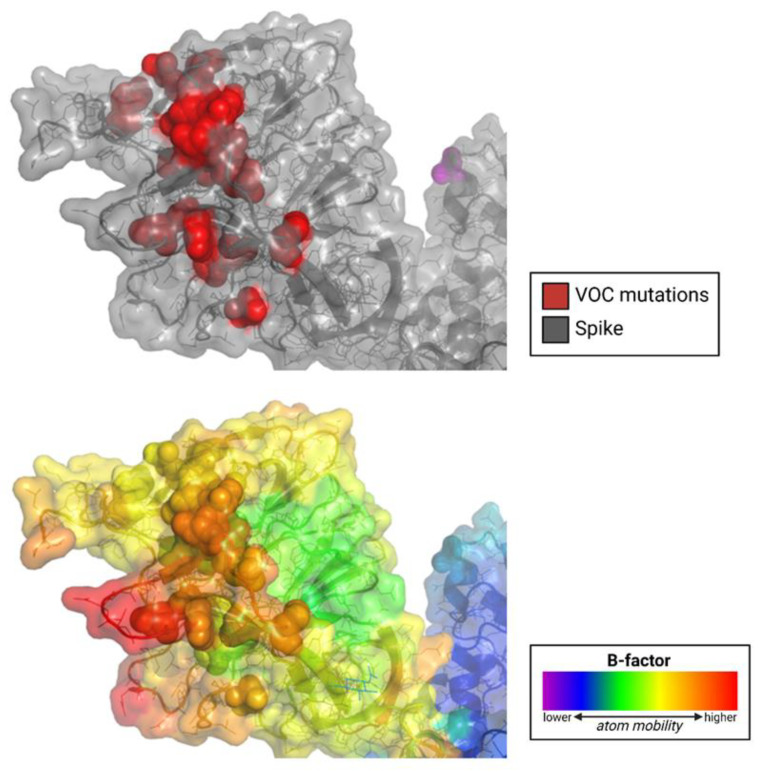
SARS-CoV-2 variants of concern (VOC) acquire mutations in the N-terminal domain (NTD) at the sites of the highest flexibility. The cryo-EM structure of the prefusion spike glycoprotein is shown. Top panel, the NTD is shown in gray; mutations in variants of concern are shown in red; bottom panel, the NTD is colored by B-factor, indicating structural variability.

Importantly, these mutations in the NTD occur in the most flexible part of the spike protein (Figure 2, Figure 3 and Figure 4). The NTD has a far higher B-factor than other parts of the spike, indicating its high structural variability. The functional consequence of mutations that impact flexibility is likely to improve fitness since these mutations distinguish VOCs. For example, increased flexibility of an epitope is likely to reduce antibody binding.

A separate site of importance is the S1-RBD, which has also mutated extensively in highly contagious variants of concern. Though it is known that the RBD binds to ACE2 to mediate host cell entry, several of these mutations have decreased the RBD’s affinity for ACE2 in spite of the increased transmission of more recent variants [9]. Although the functional consequence of mutations in the RBD likely contributes to immune evasion, it is possible that the RBD takes part in other interactions that affect infectivity.

The furin cleavage site (FCS) is another key to infection, as its cleavage promotes or enables virus-host cell fusion. The identity of host cell proteases that facilitate this cleavage is also of interest and is discussed further in this review.

## 2. Sugars as SARS-CoV-2 Spike Protein Ligands

### 2.1. Sialosides

As mentioned before, a dual-receptor mechanism binding to DPP4 and host sialoside sugars was proposed to be used by MERS-CoV for the infection of host cells. Awasthi et al. noted that the SARS-CoV-2 S1-NTD has three divergent loop regions that structurally resemble the MERS-CoV sialoside-binding pocket, as confirmed by a cryo-EM study [4,10]. Specifically, the β14–β15 loop of the NTD is implicated in sialoside binding [4].

Computational binding studies found that diverse sialosides interacted with and localized to the proposed sialoside-binding pocket in the NTD of both MERS-CoV and SARS-CoV-2. SARS-CoV was not found to interact similarly with sialosides, demonstrating binding in various locations of the NTD, which was the expected result as SARS-CoV is not known to bind to sialosides [4]. Further, Milanetti et al. also predicted a sialoside-binding pocket in the SARS-CoV-2 NTD by surface iso-electron density mapping [11].

Awasthi et al. also propose that the predicted ability of SARS-CoV-2 to engage diverse sialosides could explain its high infectivity with broad tissue tropism [4]. The distribution of sialosides in the respiratory tract could explain why SARS-CoV-2 exhibits high infectivity for these cells despite their limited ACE2 expression.

Another proposed function of sialoside binding is the facilitation of viral surfing over the host cell surface [11,12,13,14]. Viral surfing describes the movement of SARS-CoV-2 virions across a cell surface’s sialic acid layer to find and attach to ACE2. Similarly, Milanetti et al. proposed a dual or even triple binding of SARS-CoV-2 to ACE2 and gangliosides present in lipid rafts [11]. A glycan-binding domain could bind to certain glycosphingolipids present in lipid rafts. Lipid raft coalescence is proposed to lead to the recruitment of ACE2, and ganglioside expression is higher in epithelial intestinal and brain cells, supporting the potential role of gangliosides in SARS-CoV-2 infectivity.

Sialoglycan microarrays were used to test the spike protein’s sialic acid binding capacity. Hao et al. did not determine significant fluorescent signals when recombinant SARS-CoV-2 S protein was incubated with sialic acid-containing oligosaccharides on an array chip [15]. Though, these immobilized sialic acids may not model the native presentation of sialic acids on the cell surface in vivo, where sialic acids cluster in the flexible plasma membrane. Baker et al. used polymer-stabilized gold nanoparticle bearing sialic acids to confirm the binding of single sialic acid to the S protein, but not sialyllactoses [16].

Yang et al. investigated the role of glycans containing sialic acids on the ACE2 receptor for SARS-CoV-2 infection [17]. They found that glycans did not greatly contribute to the binding of the S protein to ACE2 and that ACE2′s sialic acids actually shielded cells from pseudovirus binding. Chu et al. then treated epithelial cells with neuraminidase to remove cell surface sialic acids [18]. They found that this treatment reduced MERS-CoV entry by 86%, which was expected since MERS-CoV uses DPP4 and sialic acids as coreceptors to facilitate binding. However, this sialic acid-removing treatment increased SARS-CoV and SARS-CoV-2 infection by 492% and 80.3%, respectively [19]. These results indicate that the presence of cell-surface sialic acids prevents ACE2-S protein binding, thus inhibiting virus entry.

### 2.2. Gangliosides

Though Yang et al. and Chu et al. reveal a decreasing likelihood that sialic acids potentiate SARS-CoV-2-cell binding, a preprint study by Nguyen et al. suggested that the RBD had an affinity for monosialylated gangliosides [17,18,20]. Using an artificial membrane embedded within gangliosides, they found that three monosialylated gangliosides (GM1, GM2, and GM3) were recognized by the RBD [20]. The affinity was similar to that of the glycan heparan sulfate, another proposed ligand of the RBD. Next, by depleting cell surface sialic acids using sialyltransferase inhibition, genetic knockout of sialic acid biosynthesis, and neuraminidase treatment of ACE2-expressing cells, they demonstrated decreased binding and infection.

Functions of gangliosides supporting their theoretical implication in SARS-CoV-2 infection include their negatively charged flat surface that attracts the electropositive tip of virus envelope proteins, ability to facilitate the recruitment of virus protein receptors from lipid rafts, association with cholesterol to form lipid rafts that could enhance fusion and activation of viral proteins through membrane chaperone properties [19].

Fantini et al. proposed a ganglioside binding domain at the top of the NTD, positing that it allows the S protein to interact with lipid rafts independently of the RBD [21]. The study found that neutralizing antibodies directed against the NTD’s tip did, in fact, prevent access of the S protein to lipid rafts independently of RBD-ACE2 interactions [21]. Though, Fantini et al.’s data led to the proposal that hydroxychloroquine and other antimalarials could block the interaction between the S protein and cell surface gangliosides due to their affinity for gangliosides. The long-term lack of clinical validation for this strategy is not supportive of this theory [22].

### 2.3. Heparan Sulfate

Heparan sulfate is a highly conserved, negatively charged linear polysaccharide and cellular receptor found in almost all mammalian cells. In its proteoglycan form, HS binds to a variety of extracellular proteins, with wide-ranging functions related to development, inflammation, coagulation, angiogenesis, and viral entry. Heparan sulfate proteoglycans (HSPGs) are known to serve as coreceptors for many viruses [23].

Clausen et al. determined via molecular docking that the RBD likely contains a positively charged site adjacent to the ACE2-binding site that binds negatively charged heparan sulfate [24]. Subsequent competition studies, enzymatic removal of HS, and genetic studies showed that recombinant and pseudoviral S proteins, as well as authentic SARS-CoV-2 virions, bind to cell surface HS cooperatively with ACE2 [24].

A ternary complex of ACE2, heparin and the S protein using heparin as a scaffold was observed by Clausen et al. in vitro. Kearns et al. demonstrated a polyanionic HS-binding site that starts at the RBD and runs between the RBD and NTD down to the furin cleavage site [25]. Electron micrographs suggested that binding to heparin enhances the open conformation of the RBD, which supports ACE2 binding. Further, in vitro treatment of cells with heparin lyases that degrade HS significantly reduced infection. Clausen et al. concluded that HS not only supports infection but is an essential factor in infection that enables the RBD to bind ACE2. This proposed role has since been supported by a few studies (including Liu et al. [26]).

HSPGs have previously been proposed as coreceptors for HCoV-NL63 [27] and SARS-CoV [28]. In both cases, HSPGs were also proposed as necessary adhesion molecules for HCoV-NL63 and SARS-CoV infection. In fact, the basis of treatment of SARS with lactoferrin, an innate immunity protein, is lactoferrin’s colocalization with HSPGs, blocking S protein-HSPG binding, and thus infection [28,29]. Also, HS’s role in coagulation could be responsible for thrombotic complications seen in critically ill COVID-19 patients since the S protein could outcompete antithrombin and heparin cofactor II for HS binding [30].

### 2.4. Summary of Sugars as SARS-CoV-2 Spike Protein Receptors

Experimental data has yet to reveal anything conclusive regarding the role of sialosides or gangliosides in SARS-CoV-2-cell binding. Studies offer conflicting data regarding the enabling or prevention of virus entry due to these cell surface sugars. Due to the structural basis for the spike protein’s proposed affinity for sialosides, including divergent loop regions in the NTD resembling sialoside-binding pockets and locations with an affinity for gangliosides in the NTD and RBD, more investigation into spike-sialoside interactions is warranted. If sialosides or gangliosides do, in fact, play a role in coronavirus host-cell binding, it is likely to use a novel mechanism yet unknown.

More likely is the possibility of heparan sulfate and its proteoglycan form as an important coreceptor for SARS-CoV-2. Strong but limited experimental data supports its role as a necessary or supporting factor for cell-virus adhesion, as its binding to the S protein prompts the open conformation of the RBD to accommodate subsequent ACE2 binding and infection [17].

## 3. Other Molecules

### 3.1. LRRC15 as a SARS-CoV-2 Spike Protein Ligand

Leucine-rich repeat (LRR) proteins are a family of functionally unrelated α/β horseshoe-shaped proteins that contain tandem repeats of 20–30 amino acids with an unusually high composition of leucine [31]. Leucine-rich repeat-containing protein 15 (LRRC15) is a member of the LRR family with many known functions, including innate immunity and nervous system development [32]. LRRC15 is known to be involved in the negative regulation of protein localization to the plasma membrane [33].

**Figure 5:**
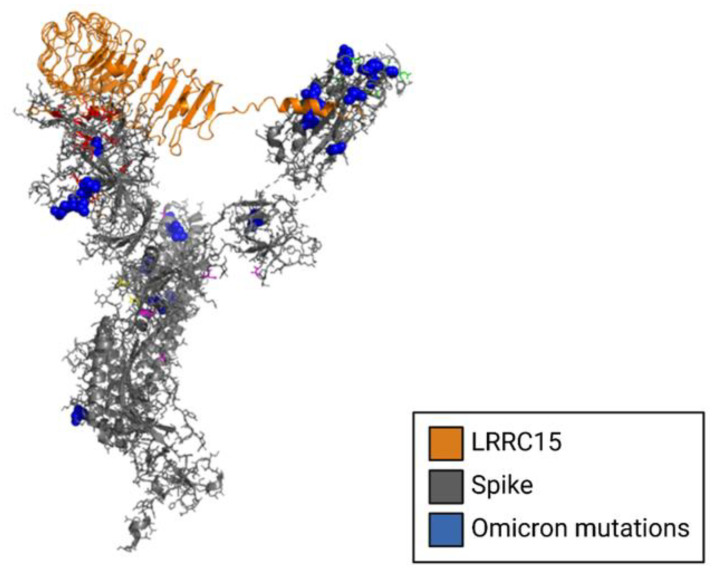
Structural model of LRRC15 complexed to the NTD of the SARS-CoV-2 spike glycoprotein. The cryo-EM structure of the spike protein is shown in gray complexed to LRRC15, shown in gold. Spike mutations from the omicron variant are shown in blue.

Multiple late-2021 preprints independently identified LRRC15 as a ligand of the SARS-CoV-2 spike protein (Figure 5). Shilts et al. employed two strategies to determine possible ligands of the spike protein [34]. First, HEK293 cells were individually transfected with one of 2363 genes encoding cell surface membrane proteins, measuring protein-spike binding with flow cytometry. Second, a genome-wide CRISPR activation library was used in RPE1 cells to identify which genes, when upregulated, induced the binding of the spike protein. Both systematic cell-based screens identified LRRC15 as SARS-CoV-2 spike protein ligands, though structural details of this interaction were unclear [34].

Loo et al. also identified LRRC15 as a spike protein ligand using a CRISPR activation strategy similar to Shilts et al. [34,35]. Further, LRRC15 sequestered virions and functioned as a negative receptor that suppressed live SARS-CoV-2 infection in trans [35]. Since LRRC15 and ACE2 expression are mutually exclusive (not expressed in the same cell types) in lung cells, it follows that LRRC15′s virion sequestration may prevent virions from reaching ACE2-expressing cells.

Additionally, LRRC15 expression could regulate the reaction of fibroblasts to infection. It is found in collagen-producing myofibroblasts, where it regulates collagen production. Since it is known to be upregulated by proinflammatory cytokines, including TNFα, IL-1β, and IFNγ [36], LRRC15 could suppress lung fibrosis during virus-induced inflammation, independent of virion sequestration and immobilization. LRRC15 could potentially help fibroblasts transport virions to antigen-presenting cells.

A subsequent decrease in LRRC15 levels following inflammation could promote collagen production to support lung repair. It is posited by Loo et al. that dysregulation of this system caused by chronic lung infection could cause inappropriate collagen production, contributing to lung fibrosis seen in “long-haul” COVID patients [35].

Interestingly, when compared to the earlier D614G variant of SARS-CoV-2, Loo et al. found that the mutations in the Delta variant’s spike protein reduced LRRC15′s antiviral activity. This indicates that the numerous mutations of the spike protein in highly contagious variants could be attributed to an adaptation against the anti-SARS-CoV-2 activity of LRRC15.

A third preprint, by Song et al., affirmed the *trans*-inhibition of viral entry of SARS-CoV-2 by LRRC15 [37]. The interaction of LRRC15 with the RBD was not found to compete with the interaction of ACE2 with the RBD. LRRC15 did, in fact, inhibit spike-mediated viral entry in LRRC15-expressing ACE2-negative cells as well as neighboring ACE2-positive cells in trans. LRRC15 had a specific inhibitory effect on multiple variants of SARS-CoV-2 and SARS-CoV-1 but not MERS-CoV, which Song et al. noted as a suggestion of an evolutionary arms race between humans and coronaviruses. Song et al. also confirmed by analysis of human lung single cell RNA sequencing that LRRC15 expression is primarily detected in fibroblasts, including those pathologically enriched in COVID-19 patients.

### 3.2. Summary of LRRC15 Relating to SARS-CoV-2

These preprints display convincing experimental evidence that LRRC15 binds to the spike protein, offering mechanistic theories with plausibility established by experimental observations. The role of LRRC15 is currently best explained as SARS-CoV-2-virion-sequestering, protecting nearby ACE2-positive cells from infection. Further speculation reveals a possible mechanism by which LRRC15′s role in collagen production regulation on fibroblasts could contribute to “long-haul” COVID. These roles have earned the protein its characterization as a “master regulator” of SARS-CoV-2 infection and collagen production associated with “long-haul” COVID-related lung fibrosis.

More experimental investigation into the spike-LRRC15 interaction is warranted to further establish the theories about its potential role in preventing infection. Resolving the structure of the spike-LRRC15 complex could offer insight into the exact binding location of LRRC15 on the spike protein.

### 3.3. Neuropilin-1 as a SARS-CoV-2 Spike Protein Ligand

Neuropilin-1 is a glycoprotein receptor found in neurons that have functions related to angiogenesis, neuronal development, and immune response regulation [38].

Some studies have identified NRP-1 as a coreceptor for SARS-CoV-2 entry [38,39,40]. The presence of NRP-1 on the host cell membrane has been shown to increase infection and spread of SARS-CoV-2, as shown by Cantuti-Castelvetri et al. [39]. Particularly, NRP-1 is implicated in neurological manifestations of COVID-19, as it is thought to enable SARS-CoV-2 to enter the central nervous system through the respiratory and olfactory epithelia—where NRP-1 is highly expressed—including those of the nasal cavity.

**Figure 6:**
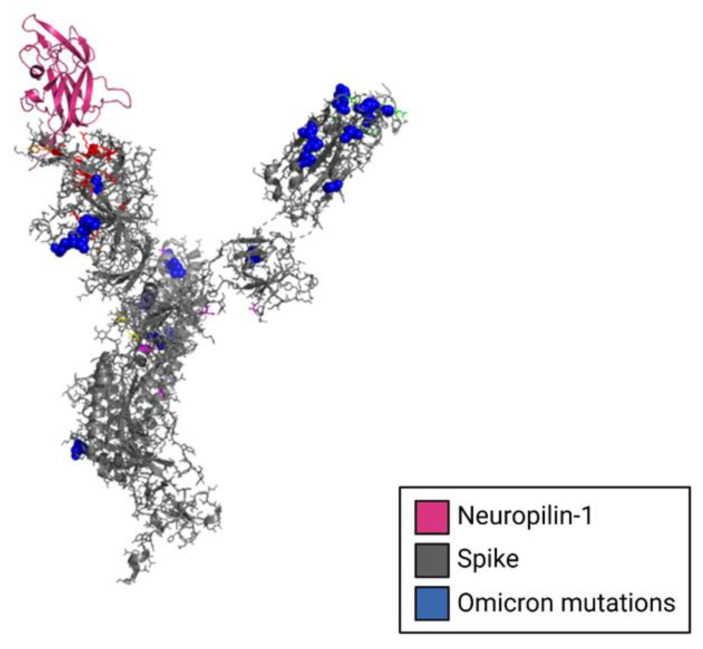
Structural model of Neuropilin-1 complexed to the NTD of the SARS-CoV-2 spike glycoprotein monomer. The cryo-EM structure of the spike protein monomer is shown in gray complex to neuropilin-1, shown in magenta. Spike mutations from the omicron variant are shown in blue.

Daly et al. explains that NRP-1 binds furin-cleaved substrates such as the one in the spike protein (Figure 6) [40]. Daly et al. successfully demonstrated that the furin-cleaved spike protein binds directly to cell surface NRP-1. Further, blocking the S1-NRP-1 interaction with small-molecule inhibitors and monoclonal antibodies reduced viral infection in vitro. This finding has significant implications for future antiviral therapeutics.

## 4. Non-Receptor Spike-Host Cell Protein Interactions

### 4.1. SARS-CoV-2 Spike Protein Cleavage and Restriction Factors

The SARS-CoV-2 spike protein relies on cleavage by proteases on the cell surface to transform into its infectious conformation that enables cell entry through plasma membrane fusion [41]. Spike protein cleavage/fusion at (or in close proximity to) the cell surface is crucial to successful infection. Spike protein fusion with host cell membranes near the cell surface permits avoidance of restriction factors located in early and late endosomes within the cell. The furin cleavage site where the spike is cleaved is thought to be an advantage of SARS-CoV-2, as viruses that lack such a cleavage site generally must enter cells through the restriction-factor-containing endosome [42].

Restriction factors are proteins that host cells use as the first line of defense against viruses [42]. Some interfere with viral replication and propagation, while others are sensors that trigger innate immune responses. They are generally induced by interferons.

Experimental evidence showed that SARS-CoV-2 is predominantly sensitive to IFITM2, a restriction factor that inhibits virus-host cell fusion [43]. The same study observed decreased infectivity when removing the furin cleavage site from pre-Omicron variants, supporting the theory that this cleavage is an advantage of the virus. Additionally, SARS-CoV-2 was found to be highly sensitive to IFN-β and IFN-γ. IFN-α and IFN-λ were less effective but still demonstrated restrictive properties in various cell types. It is not yet elucidated how changes in Omicron’s furin cleavage site (and consequently its protease-based infection cascade) may affect its interactions with restriction factors.

### 4.2. Membrane-Associated Serine Proteases

The spike protein must undergo a conformational change at the RBD to interact with ACE2. TMPRSS2 has been known as the first host cell-surface protease used by the spike protein to cleave its furin cleavage site prompting the conformational changes necessary to enter the cell [41]. Following cleavage by TMPRSS2, the spike is further cleaved and processed by furin and cathepsin, then fusing with the cell and provoking viral replication.

Currently, Omicron variants inefficiently use TMPRSS2, causing the spike to rely on cell entry through endocytosis [44]. This caused a change in the cellular tropism of SARS-CoV-2, moving away from TMPRSS2-expressing cells. However, BA.5 has recently shown efficient use of TMPRSS2, indicating a possible shift back to pre-Omicron tropism and infectious mechanism [45].

## 5. Summary

The identity of a putative SARS-CoV-2 coreceptor has yet to be determined. Though, few proposed candidates increasingly demonstrate activity as potential ligands of the spike protein (Table 1). Further experimentation is warranted to elucidate the roles of these candidates in the SARS-CoV-2 infection mechanism. Significant improvements in the prevention and treatment of COVID-19 may be achieved by understanding how to modulate the interactions between the S1-NTD/RBD and coreceptors, antibodies, and restriction factors.

## 6. Methods

### 6.1. SARS-CoV-2 Mutations in Variants of Concern

Mutations in SARS-CoV-2 variants of concern are shown with the Wuhan-Hu-1 sequence as a reference (GENBANK accession number MN908947). The Centers for Disease Control and Prevention classification and definitions of mutations that distinguish variants of concern were used: https://www.cdc.gov/coronavirus/2019-ncov/variants/variant-classifications.html (accessed on 26 April 2022) [48].

### 6.2. Mapping Mutations and Structural Variability on Structure of the Spike Protein Monomer

The prefusion SARS-CoV-2 spike glycoprotein with a single receptor-binding domain up (PDB 6VSB) [49] was used for mapping mutations and structural variability (B-factor) in variants of concern.

### 6.3. Modeling Interactions between LRRC15, Neuropilin-1 and the SARS-CoV-2 Spike Protein NTD

The crystal structure of Neuropilin-1 (PDB 7JJC) [40] was docked to the SARS-CoV-2 spike protein NTD using the program HDOCK [50]. A structural model of human LRRC15 was generated by SWISS-MODEL [51] and docked to the SARS-CoV-2 spike protein NTD using the program HDOCK [50].

## Figures and Tables

**Table 1:** Summary of potential coreceptor ligands of the SARS-CoV-2 spike protein.

Ligand	Description	Proposed Virological Function	References
Sialic acid	Sugar chain with nine-carbon backbone found on the surfaces of all vertebrate cells. Implicated in various functions and diseases.	S1-NTD may bind sialic acids in a sialosides-binding pocket that resembles that of the MERS-CoV S1-NTD. Sialic acid may act as a coreceptor to help S1 reach ACE2 via viral surfing, or it may inhibit ACE2-S1 binding.	[4,11,17,46]
Monosialylated gangliosides (GM1, GM2, GM3)	Sialic acid-containing glycosphingolipids widely expressed in the nervous system. As part of the plasma membrane’s outer leaflet, these sugar chains are key to cell-cell recognition, adhesion, and signal transduction.	Negatively charged flat surface may attract the electropositive tip of virus envelope proteins. Association with cholesterol to form lipid rafts could enhance fusion. Membrane chaperone properties could enhance activation of viral proteins.	[19,21,47]
Heparan sulfate proteoglycans (HSPGs)	Glycoprotein with attached heparan sulfate polysaccharide chains. Found as a cellular receptor in almost all mammalian cells with functions related to development, inflammation, coagulation, angiogenesis, and viral entry.	Binding between HSPGs/HS and a polyanionic path along the S1-RBD, NTD, and FCS may enhance the open conformation of the RBD, supporting RBD-ACE2 binding.	[23,24,25,26]
Leucine-rich repeat-containing protein 15 (LRRC15)	Protein family with unrelated functions and characteristic α/β-horseshoe shape with leucine-rich tandem repeats. Functions of LRRC15 include innate immunity, down regulation of protein localization to the plasma membrane, and nervous system development.	LRRC15 may sequester and immobilize SARS-CoV-2 virions. Upregulated by proinflammatory cytokines, LRRC15 could suppress lung fibrosis during virus-induced inflammation. Demonstrated affinity for S1-NTD.	[31,32,33,34,35,37]
Neuropilin-1 (NRP-1)	Glycoprotein receptor found on cell membranes of neurons with functions related to angiogenesis, neuronal development, and immune response regulation.	Potentially enables SARS-CoV-2 virions to enter the nervous system through respiratory and olfactory epithelia. Known to bind furin-cleaved substrates.	[38,39,40]

## Data Availability

Not applicable.
